# Role of inflammatory burden and treatment on joint space width in psoriatic arthritis—a high-resolution peripheral quantitative computed tomography study

**DOI:** 10.1186/s13075-023-03124-5

**Published:** 2023-08-03

**Authors:** Yingzhao Jin, Isaac T Cheng, Ho So, Dongze Wu, James F Griffith, Vivian W Hung, Ling Qin, Cheuk-Chun Szeto, Agnes WS Chan, Lai-Shan Tam

**Affiliations:** 1 Department of Medicine and Therapeutics, The Prince of Wales Hospital, The Chinese University of Hong Kong, Shatin, Hong Kong, China; 2 Li Ka Shing Institute of Health Sciences (LiHS), Faculty of Medicine, The Chinese University of Hong Kong, Hong Kong, China; 3 Department of Imaging and Interventional Radiology, The Prince of Wales Hospital, The Chinese University of Hong Kong, Hong Kong, China; 4 Bone Quality and Health Centre, Department of Orthopaedics and Traumatology, The Chinese University of Hong Kong, Hong Kong, China

**Keywords:** Psoriatic arthritis, High-resolution peripheral quantitative computed tomography, Joint space, Inflammatory burden, Treatment

## Abstract

**Background:**

To investigate the relationship between disease-related parameters and joint space width (JSW) on high-resolution peripheral quantitative computed tomography (HR-pQCT) in psoriatic arthritis (PsA) patients.

**Methods:**

PsA patients who underwent HR-pQCT examination of the second to fourth metacarpophalangeal joint (MCPJ 2–4) were recruited in this cross-sectional study. The joint space metrics included joint space volume (JSV), mean, minimum, and maximum JSW, JSW asymmetry, and distribution. Correlation analysis and multivariable linear regression models were used to determine the association between disease-related variables and JSW.

**Results:**

Sixty-seven patients [37 (55.2%) males; median (IQR) age: 57.0 (53.0, 63.0); median disease duration: 21 (16, 28) years] were included in this analysis. Multivariable linear regression analysis demonstrated that males had larger JSV (MCPJ 2–4), mean (MCPJ 4), and maximum JSW (MCPJ 3). Longer disease duration (MCPJ 2–3) and higher ESR values (MCPJ 3) were negatively associated with mean and maximum JSW, while higher damage joint count was negatively associated with mean and minimum JSW (MCPJ 2). Use of conventional synthetic disease-modifying anti-rheumatic drugs (csDMARDs) was negatively associated with minimum JSW (MCPJ 3) while use of biologic DMARDs (bDMARDs) was positively associated with minimum JSW (MCPJ 2).

**Conclusion:**

Higher inflammatory burden as reflected by longer disease duration, higher ESR levels, and damage joint count was negatively associated with mean, maximum, and minimum JSW, while suppression of inflammation using bDMARDs seems to limit the decline in JSW.

**Supplementary Information:**

The online version contains supplementary material available at 10.1186/s13075-023-03124-5.

## Background

Psoriatic arthritis (PsA) is an autoimmune inflammatory condition with diverse clinical manifestations that include peripheral and axial arthritis, enthesitis, dactylitis, skin and nail psoriasis, as well as other manifestations such as anterior uveitis and inflammatory bowel disease [1]. Joint damage, as a result of chronic inflammation often leads to bone, cartilage, and soft tissue damage which can be visualized on radiography as bone erosion and joint space narrowing (JSN). JSN, as a surrogate of cartilage damage, is more strongly associated with functional impairment than bone erosion. making it a valid target for treatment [2].

Radiography is currently the main modality used to monitor structural damage in PsA [3], enabling an assessment of bone damage (erosions), cartilage damage (scored as JSN), and ligament damage resulting in malalignment. Radiography, however, has limitations in with respect to spatial resolution, sensitivity, and responsiveness [4]. High-resolution peripheral quantitative CT (HR-pQCT) enables a detailed assessment of bone microstructure with high reproducibility in detecting bone erosions and new bone formation at the MCPJs [5]. High spatial resolution (isotropic resolution: 82 μm for XtremeCT I; 61 μm for XtremeCT II), HR-pQCT is more sensitive at detecting erosions compared to radiography and magnetic resonance imaging (MRI) [6]. A reproducible, high-throughput, robust, fully automated method for evaluating MCP joint space width (JSW) using HR-pQCT has been developed [7]. In vivo quantification of 3D joint space morphology improves early detection of joint damage in rheumatological diseases [8]. JSW measurement in RA is associated with Sharp/van der Heijde (SvdH) score in the MCPJ 2 and 3 [9], and is reliable in longitudinal studies [10]. Using HR-pQCT, we found that inflammation led to bone damage (erosions and enthesiophytes) and trabecular bone loss on the 2^nd^ and 3^rd^ metacarpal head (MCH) in PsA patients [11]. Nevertheless, the association between inflammatory burden and JSW remains unclear. Tumor necrosis factor (TNF) inhibition arrested the progression of bone erosion but not enthesiophyte formation after one year, while interleukin-17 (IL-17) inhibition arrested the progression of both bone erosion and enthesiophyte formation after six months [12]. Whether the use of disease-modifying anti-rheumatic drugs (DMARDs) in PsA patients can prevent changes in JSW remains uncertain.

We hypothesized that JSW parameters measured using HR-pQCT might be associated with disease-related variables and treatment in PsA patients. We conducted a cross-sectional HR-pQCT study of the 2–4 MCPJ in PsA patients to determine the association between demographic, clinical, and treatment variables and JSW.

## Methods

### Patients

Seventy-six consecutive clinical PsA patients who had a standardized clinical and HR-pQCT assessment were recruited between 2017 and 2018. Erosion and enthesiophyte data on 60 out of these 76 patients were previously published [13]. All PsA patients fulfilled the ClASsification for Psoriatic ARthritis (CASPAR) criteria [14]. The treatment regime, which included conventional synthetic DMARDs (csDMARDs) and biologic DMARDS (bDMARDs) was determined by the attending rheumatologist.

### Clinical assessment

Clinical and demographic parameters were recorded. Clinical assessment included the swollen, tender (66/68) joint count, deformed joint count, and the presence of dactylitis. Disease activity was assessed using the Disease Activity index for PsA (DAPSA) [15]. Clinical assessment was performed on the same day of imaging. Blood tests included C-reactive protein (CRP), and erythrocyte sedimentation rate (ESR). Health Assessment Questionnaire (HAQ) disability index was used to assess functional disability. Information regarding drug treatment including cs/bDMARDs was retrieved from the electronic management system. Patients underwent both X-ray and HR-pQCT on the same day. The joint space domain on standard radiography of MCPJ 2–4 of the non-dominant hand was scored, using the SvdH joint space score, by an experienced reader as follows: 0 = normal, 1 = asymmetrical or minimal joint space narrowing up to a maximum of 25%, 2 = definite narrowing with loss of up to 50% of the normal joint space, 3 = definite narrowing with loss of 50–99% of the normal joint space or subluxation, 4 = absence of a joint space, evidence of ankylosis, or dislocation [16].

### High-resolution peripheral quantitative CT

All patients underwent HR-pQCT examination (XtremeCT I, SCANCO Medical AG, Brüttisellen, Switzerland) of MCPJ 2–4 of the non-dominant hand. HR-pQCT scanning was performed by a single investigator, blinded to the clinical information. The patient’s forearm was immobilized in a carbon fiber cast fixed within the scanner gantry. An anteroposterior scout view was used to define the region of interest (ROI). The scan region was 107 slices distal and 325 slices proximal to the apical margin of the head of MCPJ 3. Motion artifact was evaluated using the manufacturer’s scoring system (1–5). Examinations with a motion artifact score of 4 or 5 were excluded.

### Joint space analysis

Volumetric joint space was quantified using an algorithm developed by consensus from the Study group for eXtreme Computed Tomography in Rheumatoid Arthritis (SPECTRA) [17]. Images in the coronal and sagittal planes (2-dimensional) were automatically reconstructed for analysis. A rheumatologist with HR-pQCT expertise graded the degree of subluxation (none, subluxation, dislocation) and bone-on-bone contact (yes, no). 3D joint space volume (JSV, mm^3^), maximum (Max), mean (Mean), minimum (Min) JSW (mm), JSW SD (mm), and asymmetry (Asym, defined as JSW.Max/JSW.Min ratio] were calculated.

### Statistical analysis

Data are expressed as mean ± SD or median (interquartile range) for numeric variables. Descriptive statistics were used for demographic and clinical variables including frequency, mean and standard deviation, median and interquartile range. A generalized estimating equation (GEE) was used to estimate the ability of HR-pQCT to predict SvdH scores. Correlation analysis was used to determine the association of demographic (age, sex), disease-specific parameters (disease duration, tender and swollen joint count, damage joint count, DAPSA, HAQ, ESR, and CRP levels), and treatment (csDMARDs and bDMARDs) with HR-pQCT joint space parameters. Variables associated with JSW were assessed using multivariable linear regression models with adjustment for covariates (including all variables associated with JSW in the univariate analyses with a *p*-value < 0.05). All statistical analyses were performed using IBM SPSS statistics version 22.0 (SPSS, Armonk, NY, USA). Two-sided *P* < 0.05 was considered statistically significant.

## Results

### Patient characteristics

Seven patients were excluded due to HR-pQCT motion artifact and 2 patients were excluded because the algorithm could not correctly define the joint space. The images of 67 patients [37 (55.2%) males; median (IQR) age: 57.0 (53.0, 63.0)] were included in the analysis (Table 1). Despite a long disease duration (median disease duration: 21 (16, 28) years], most patients had low disease activity [DAPSA: (10.1 ± 6.6); CRP: 1.9 (0.8, 4.9) mg/L; ESR: 24 (14, 41) mm/hr], and minimal functional impairment (HAQ: 0.25 [0.00, 0.88)). Among the studied cases, 7 (10.4%) did not receive any therapy, 33 cases (49.3%) were treated with csDMARDs monotherapy, 4 cases (6.0%) were prescribed bDMARDs monotherapy, and 23 cases (34.3%) received combination therapy. The duration of the disease since the initiation of csDMARD treatment was 12.2 ± 7.8 years, while the duration of the disease since the start of bDMARD treatment was 11.9 ± 6.8 years.

**Table 1:** Demographic and clinical characteristics of patients with psoriatic arthritis

	PsA patients
Patient *N*	67
Age, year	57.0 (53.0, 63.0)
Female (%)	30 (44.8%)
Disease duration, year	21.0 (16.0, 28.0)
CRP, mg/L	1.9 (0.8, 4.9)
ESR, mm/h	24 (14, 41)
DAPSA	10.13 ± 6.55
DAPSA category	
Remission	14 (20.9%)
Low disease activity	33 (49.3%)
Moderate disease activity	18 (26.9%)
High disease activity	2 (3.0%)
HAQ	0.25 (0.00, 0.88)
Tender joint count	0 (0, 2)
Swollen joint count	0 (0, 1)
Damage joint count	4 (2, 10)
Current treatment	
csDMARDs (%)	56 (83.6%)
bDMARDs (%)	27 (40.3%)

Values are presented as mean ± SD or median (interquartile range) for continuous data and number (percentage) for categoric data

*PsA* Psoriatic arthritis, *CRP* C-reactive protein, *ESR* Erythrocyte sedimentation rate, *DAPSA* Disease Activity in Psoriatic Arthritis, *HAQ* Health Assessment Questionnaire score, *csDMARDs* Conventional synthetic disease-modifying anti-rheumatic drugs, *bDMARDs* Biologic disease-modifying anti-rheumatic drugs

Eleven MCPJ 2, 9 MCPJ 3, and 7 MCPJ 4 were excluded from further analysis due to motion artifact. The final dataset included 174 joints (56 MCPJ 2, 58 MCPJ 3, and 60 MCPJ 4). Individual MCP joint space parameters were presented in Supplementary Table 1. Out of all the MCPJ that were scanned, 3 were found to be tender and 1 was swollen at the time of scanning. However, the presence of tenderness or swelling in the joints examined using HR-pQCT did not correlate with any of the JS parameters.

### Correlation between HR-pQCT derived JSW and SvdH from radiographs

**Fig. 1:**
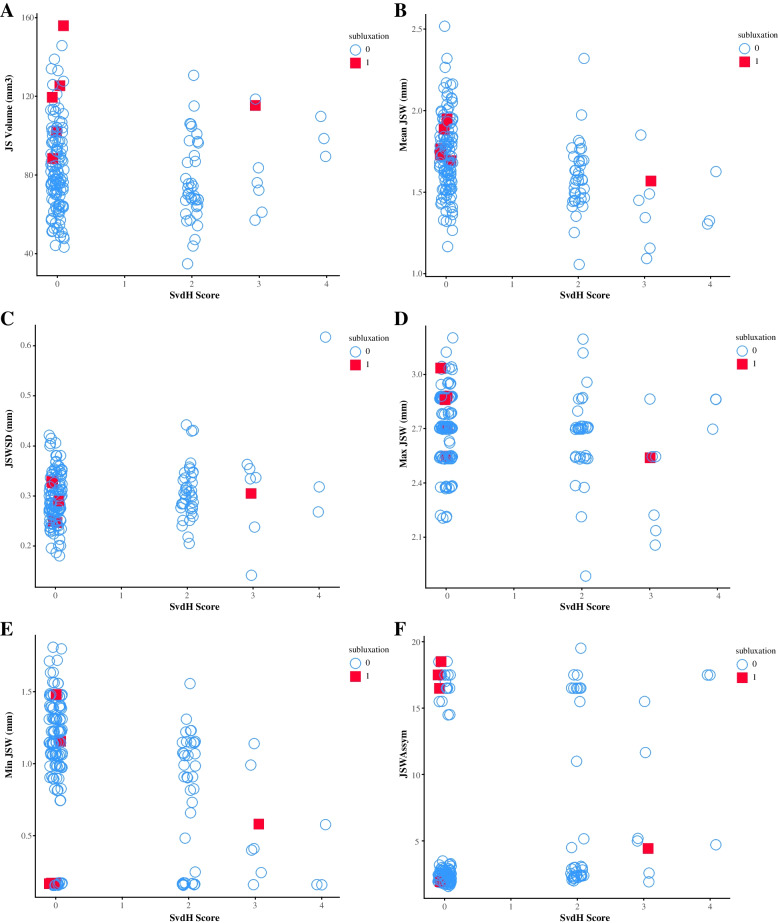
Comparison between joint space outcomes derived from HR-pQCT and van der Heijde-modified Sharp joint space scores (SvdH) from conventional radiographs for the MCPJ 2–4. **A** JS Volume; **B** Mean JSW; **C** JSW SD; **D** Max JSW; **E** Min JSW; **F** JSW Assym. Subluxations were detected more frequently by HR-pQCT than radiograph. HR-pQCT, high-resolution peripheral quantitative computed tomography; MCPJ, metacarpophalangeal joint; JS, joint space; JSW, joint space width; SD, standard deviation; Max JSW, maximum joint space width; Min JSW, minimum joint space width; Assym, joint asymmetry

Distribution of SvdH second, third, and fourth MCPJ scores is shown in Supplementary Table 2. Most patients had a SvdH score of 0 (normal) or 2 (> 50% narrowing of original JS). HR-pQCT-derived joint space parameters compared with radiographic-derived SvdH scores for MCPJ 2–4 are shown in Fig. 1. Subluxations were detected more frequently by HR-pQCT than radiographs (5 subluxations with SvdH scores 0 and 1 subluxation with SvdH score 3). GEE results indicated that a more severe (i.e., higher) SvdH score was negatively associated with a lower mean, Max and Min JSW; and higher JSW SD and Asymm (all *p* < 0.05) (Supplementary Table 3). No significant relationship was observed between the SvdH score and HR-pQCT-derived JSV (*p* > 0.05). Higher damage joint count was associated with higher SvdH score at MCPJ 2–4 (*p* < 0.05) (Supplementary Table 4).

### Univariate and multivariable analysis of disease-related parameters and HR-pQCT derived JSW and JSV

**Fig. 2:**
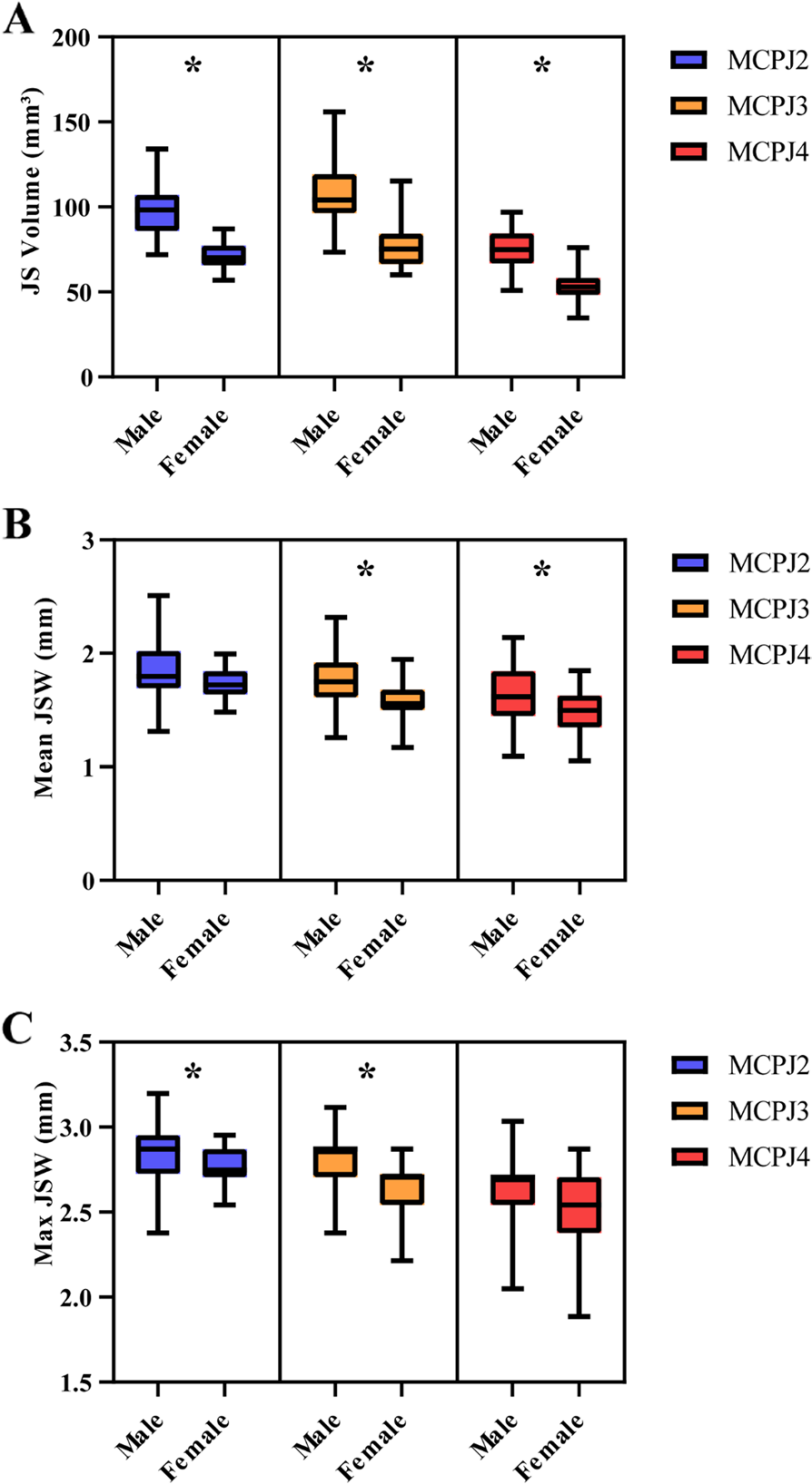
Differences in joint space parameters according to sex. *p < 0.05. **A** JS Volume; **B** Mean JSW; **C** Max Jsw. Male sex was associated with a larger JSV (MCPJ 2–4), higher Mean (MCPJ 3–4), and Max JSW (MCPJ 2–3). MCPJ, metacarpophalangeal; JS, joint space; JSW, joint space width; Max JSW, maximum joint space width

**Fig. 3:**
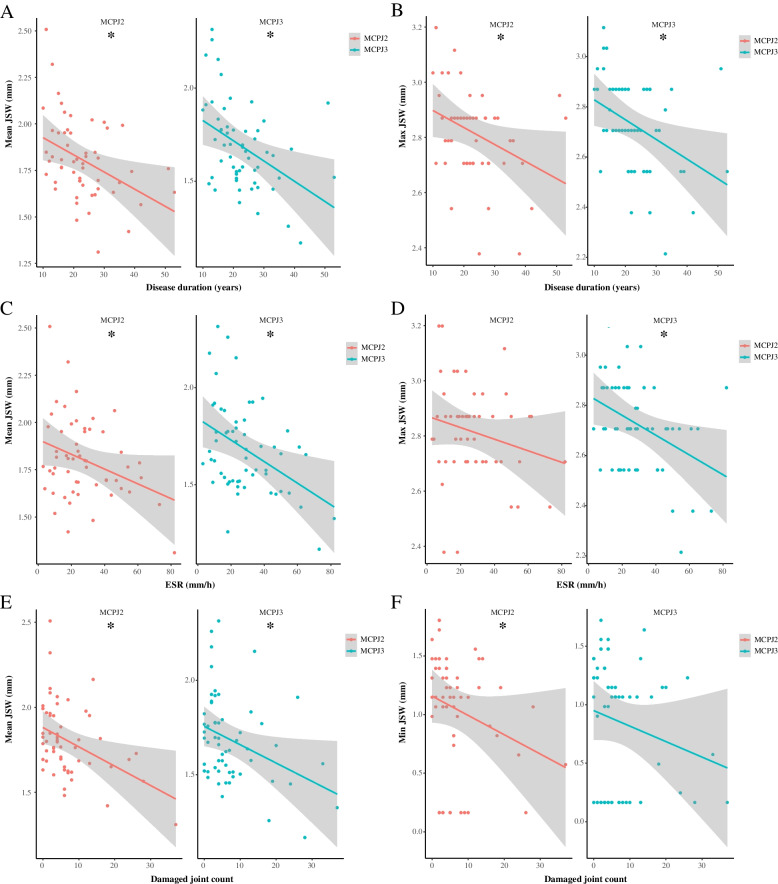
Correlation between disease-related variables and joint space parameters. *p < 0.05. **A** Correlation between disease duration and Mean JSW; **B** correlation between disease duration and Max JSW; **C** correlation between ESR and Mean JSW; **D** correlation between ESR and Max JSW; **E** Correlation between damaged joint count and Mean JSW; **F** Correlation between damaged joint count and Min JSW. Mean JSW (MCPJ 2–3) was negatively correlated with disease duration, ESR level, and damage joint count, while Max JSW was negatively correlated with disease duration (MCPJ 2–3) and ESR level (MCPJ 3), Min JSW was negatively correlated with damage joint count (MCPJ 2). MCPJ, metacarpophalangeal joint; ESR, erythrocyte sedimentation rate; JSW, joint space width; Max JSW, maximum joint space width; Min JSW, minimum joint space width

**Fig. 4:**
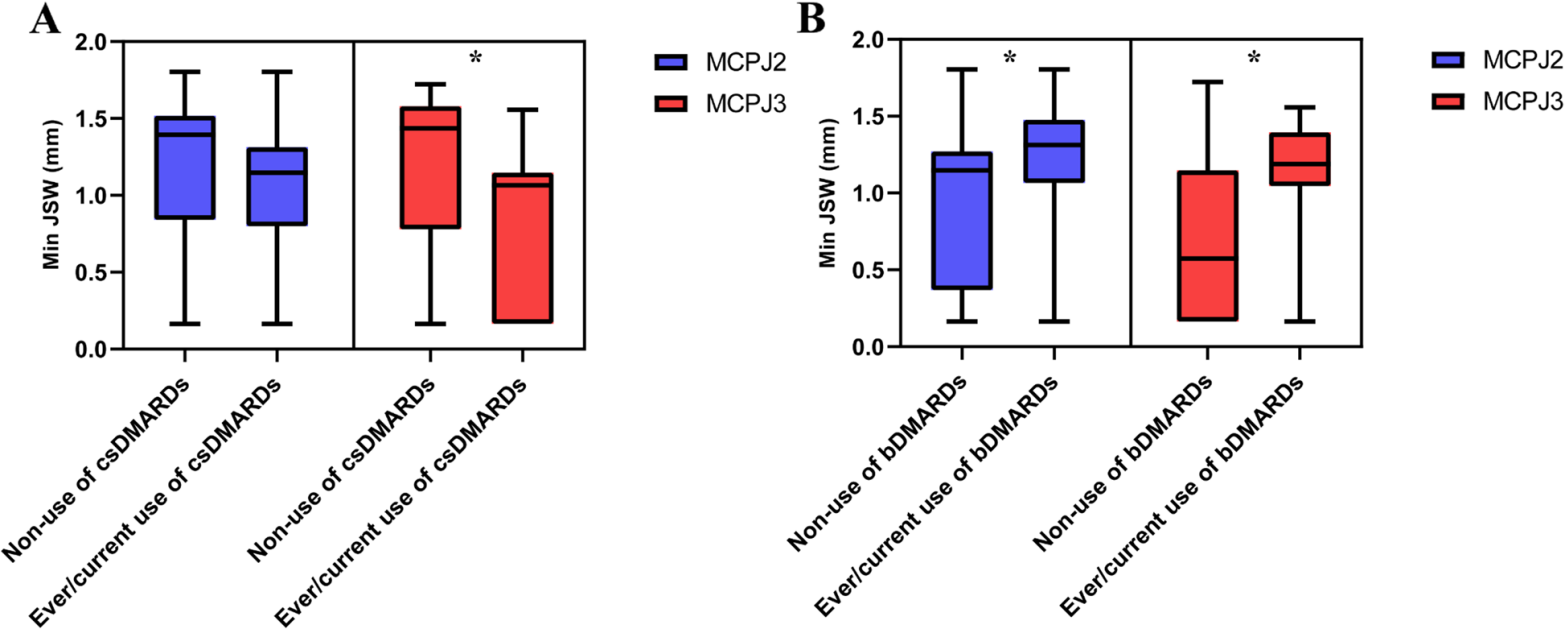
Differences in minimum JSW according to treatment. *p < 0.05. **A** csDMARDs; **B** bDMARDs. Patients receiving csDMARDs (MCPJ 3) and bDMARDs (MCPJ 2–3) had significantly lower and higher Min JSW respectively, compared to non-users. JSW, joint space width; MCPJ, metacarpophalangeal; csDMARDs, conventional synthetic disease-modifying anti-rheumatic drugs; bDMARDs, biologic disease-modifying anti-rheumatic drugs

Supplementary Tables 5–10 summarize the univariate and multivariate analysis of clinical variables, JSV, and JSW parameters. In univariate analysis, males had larger JSV (MCPJ 2–4), higher Mean (MCPJ 3–4), and Max JSW (MCPJ 2–3) (Supplementary Tables 5–7, Fig. 2). Mean JSW (MCPJ 2–3) was negatively correlated with disease duration, ESR level, and damage joint count (Fig. 3 and Supplementary Table 6). Max JSW was negatively correlated with disease duration (MCPJ 2–3) and ESR level (MCPJ 3) while Min JSW was negatively correlated with damage joint count (MCPJ 2) (Fig. 3 and Supplementary Tables 7–8). CRP level was negatively associated with Max JSW (MCPJ 2 and 4) and Min JSW (MCPJ 4), while positively correlated with JSW AS (MCPJ 4) (Supplementary Tables 7, 8, 10). Patients receiving csDMARDs (MCPJ 3) had lower Min JSW compared to non-users (*p* = 0.026), while patients receiving bDMARDs (MCPJ 2–3) had higher Min JSW compared to non-users (*p* = 0.033 and 0.008, respectively) (Fig. 4 and Supplementary Table 8).

**Fig. 5:**
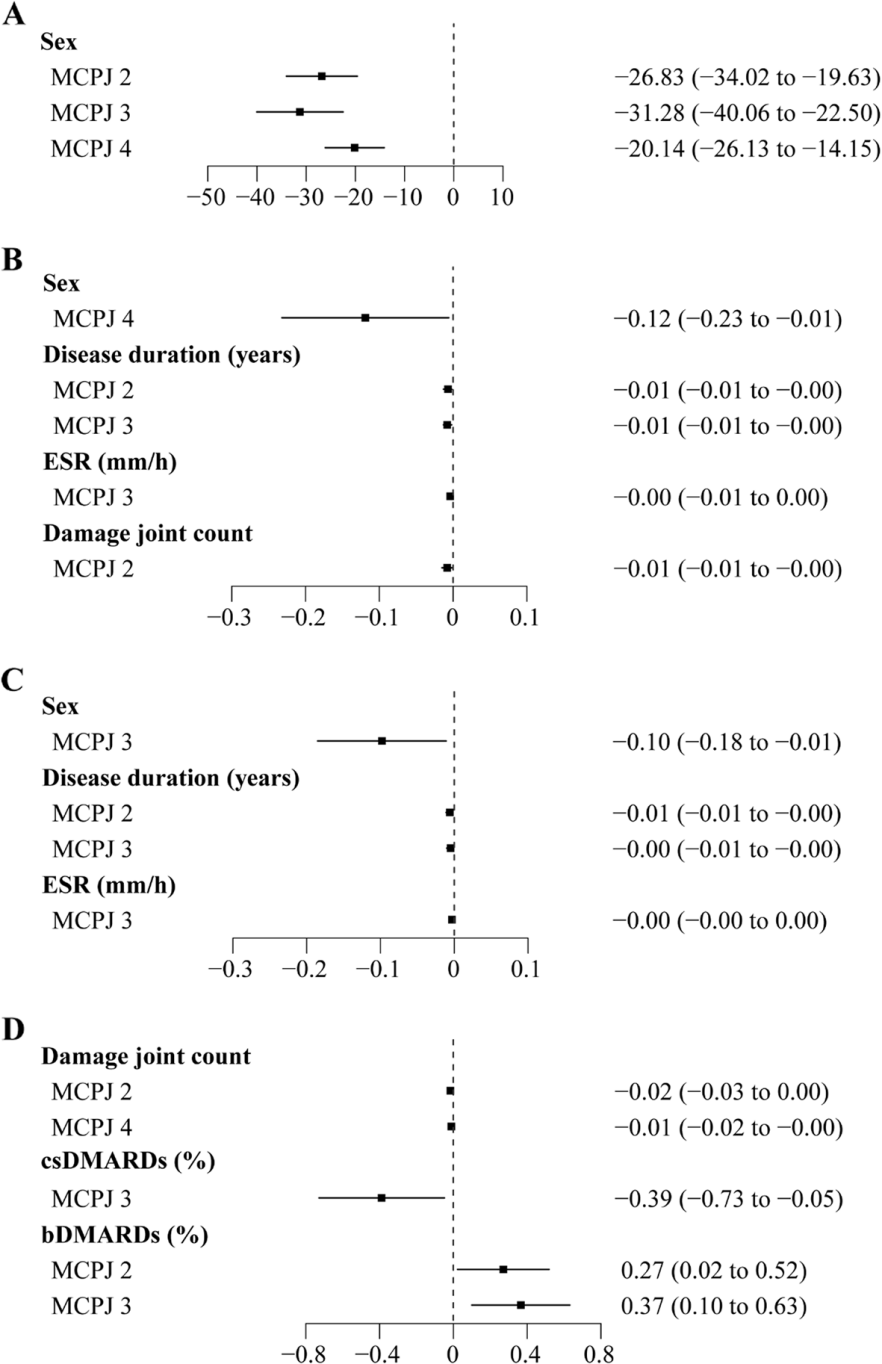
Forest plot demonstrating multivariable linear regression analysis between demographic and disease-related variables and JSW parameters at MCPJ 2 to 4. **A** Joint space volume; **B** mean joint space width (JSW); **C** maximum JSW; **D** minimum JSW. Male sex was associated with a larger JSV, higher Mean JSW (MCPJ 4), and Max JSW (MCPJ 3). Mean and Max JSW were negatively correlated with disease duration (MCPJ 2 and 3) and ESR level (MCPJ 3). Mean JSW (MCPJ 2) and Min JSW (MCPJ 2 and 4) were negatively correlated with damage joint count. Patients receiving csDMARDs (MCPJ 3) and bDMARDs (MCPJ 2 and 3) had significantly lower and higher Min JSW, respectively, compared to non-users. MCPJ, metacarpophalangeal joint; ESR, erythrocyte sedimentation rate; csDMARDs, conventional synthetic disease-modifying anti-rheumatic drugs; bDMARDs, biologic disease-modifying anti-rheumatic drugs

Supplementary Table 11 and Fig. 5 summarize the multivariate analysis of clinical variables, JSV, and JSW parameters. Multivariable regression analysis confirmed that males had larger JSV (MCPJ 2–4, β ranged from − 31.2 to − 20.1, *p* < 0.001), higher Mean JSW (MCPJ 4, *β* =  − 0.119 [95% CI: − 0.232, − 0.006], *p* = 0.039) and Max JSW (MCPJ 3, *β* =  − 0.098 [95% CI: − 0.185, − 0.011], *p* = 0.029). Mean and Max JSW were negatively correlated with disease duration (MCPJ 2 and 3, *β* ranged from − 0.08 to − 0.05, *p* < 0.05) and ESR level (MCPJ 3, *β* ranged from − 0.04 to − 0.03, *p* < 0.05). Mean JSW (MCPJ 2, *β* =  − 0.008 [95% CI: − 0.015, − 0.001], *p* = 0.034) and Min JSW (MCPJ 2 and 4, *β* ranged from − 0.016 to − 0.011, *p* < 0.05) were negatively correlated with damage joint count. Patients receiving csDMARDs (MCPJ 3, *β* =  − 0.389 [95% CI: − 0.728, − 0.049], *p* = 0.026) had significantly lower and bDMARDs (MCPJ 2 and 3, *β* ranged from 0.27 to − 0.37, *p* < 0.05) significantly higher Min JSW compared to non-users. The use of bDMARDs was also negatively associated with JSW Asymm at MCPJ 3 (*β* =  − 4.776 [95% CI: − 8.234, − 1.317], *p* = 0.008) (Supplementary Table 11). Higher HAQ score was associated with larger JSW SD at MCPJ 4 (*β* = 0.028 [95% CI:0.004, 0.052], *p* = 0.023). The number of tender and swollen joints (out of the 66/68 joints, and those MCPJ scanned by HR-pQCT which were tender or swollen at the time of assessment), DAPSA, monotherapy/combination therapy and duration of the disease since the start of cs/bDMARDs treatment were not associated with JSV or JSW parameters.

There was no significant association between the presence of anemia/osteoarthritis or the severity of enthesitis/psoriasis with the duration of disease/ESR level/presence of joint damage or treatment with biologics.

## Discussion

This is the first study to investigate the usefulness of HR-pQCT JSV and JSW parameters in PsA patients. A validated, JSW analytic HR-pQCT method was used enabling the precise determination of MCP joint space parameters in PsA. Decreased Min JSW, increased JSW SD and Asymm were associated with increasing SvdH score, similar to this seen in RA patients [18]. In addition, we found that decreased Mean and Max JSW was associated with more severe SvdH scores.

Similar to that found in RA patients, female PsA patients had smaller JSV, Mean JSW, and Max JSW compared to males [19], most likely reflecting the greater body habitus of male patients. This gender effect on JSW may need to be considered when planning HR-pQCT studies.

The ability of HR-PQCT to detect joint space narrowing in patients with a zero (Normal) SvdH score and the higher frequency of MCPJ subluxations evident on HR-pQCT, suggests that this high-resolution imaging technique can detect early joint damage and is, a result, likely to be more sensitive than standard radiography in monitoring disease progress in PsA patients. Similar to that seen in RA patients, because of the wide inherent variability in population JSW size, it is difficult to determine disease progression in PsA patients based on a single quantitative HR-pQCT outcome, such as JSV.

PsA-associated enthesitis and synovitis may lead to bone erosions and cartilage loss. As a surrogate marker of cumulative inflammatory burden, longer disease duration was associated with a lower Mean and Max JSW in both MCPJ 2 and 3. Similarly, a higher damaged joint count was associated with a lower Mean JSW (MCPJ 2) and Min JSW (MCPJ 2 and 4) in multivariate analysis. There is, as expected, a close association between radiographic and clinical joint damage. In PsA patients, radiological joint damage may precede clinical evidence of joint damage [20].

Higher HAQ score was associated with a larger JSW SD at MCPJ 4. The observed results suggested that greater functional impairment, as indicated by a higher HAQ score, may be linked to increased JSW SD indicating altered joint space morphology at the MCPJ 4. Higher HAQ scores reflect more severe joint disease and greater joint dysfunction. Patients with more advanced diseases may experience increased joint damage and subsequent larger joint space SD.

Clinical inflammation usually precedes joint damage. Radiological damage tends to be closely related to an increase in swollen joint count [20, 21]. In the current study, ESR was negatively associated with Mean and Max JSW (MCPJ 4), supporting the role of inflammation and joint damage. That said, a snapshot assessment of joint inflammation, such as the tender and swollen joint count in a cross-sectional study, may not be representative of inflammatory burden over time. In the current study, tender and swollen joint count was not associated with JSW parameters. Swollen joint count correlated with Mean, Max, and Min JSW only in the univariate analysis. This is not surprising as swollen joints will be distended with synovial fluid and joint space width may be directly reflective of articular cartilage thinning. Similarly, when inflammation subsides, the joints will become less distended, giving rise to an apparent increase in joint space narrowing which may simulate progressive cartilage thinning even though no further cartilage thinning has occurred. This is one of the limitations of using JSW as a marker of cartilage thinning and joint dame in swollen joints.

The ever/current-use of csDMARDs was associated with more severe joint space narrowing (i.e., a lower Min JSW). Radiographic progression occurred in 89% of patients treated with csDMARDs, with erosion progression and JSN progression occurring in 61% and 86% of patients, respectively [22]. In contrast, the ever/current-use of bDMARDs was associated with less severe JSN indicating that bDMARDs may be able to prevent cartilage damage. This is in agreement with a recent meta-analysis highlighting how biologic agents may retard radiographic progression in PsA patients compared to placebo [23]. This finding suggests that Min JSW may be a more useful measure of joint space than other HR-pQCT joint space parameters when monitoring treatment response.

The main limitation of this study is the cross-sectional design, which limited the assessment of the effect of disease-specific variables on joint space parameters. Also, all PsA patients in our study had long-standing disease, so we were not able to investigate the usefulness of JSW analysis in detecting subtle joint damage in PsA patients with early disease. Thirdly, although we did not find any association between potential comorbidities (e.g., anemia or osteoarthritis), or severity of enthesitis or psoriasis, with variables significantly associated with HR-pQCT parameters (e.g., duration of disease, ESR level, presence of joint damage or treatment with biologics), we did not collect data on hypergammaglobulinemia. The effects of which would need to be addressed in future studies.

## Conclusions

In conclusion, HR-pQCT JSW parameters are more sensitive than radiographic JSN parameters in detecting joint damage in PsA patients. JSW parameters correlated with inflammatory burden while less severe joint space narrowing was seen in patients treated with bDMARDs. Nonetheless, prospective studies comparing changes in JSW parameters vs JSN on radiograph before and after treatment are needed to confirm the utility of this novel assessment tool.

### Supplementary Information

**Additional file 1: Supplementary Table 1.** Joint space analysis using HR-pQCT for PsA patients. **Supplementary Table 2.** Joint space narrowing scored using the van der Heijde-modified Sharp scoring system on conventional radiographs. **Supplementary Table 3.** The ability of HR-pQCT joint space analysis to predict SvdH scores on conventional radiography using Generalized estimating equations (GEE). **Supplementary Table 4.** Univariate and multivariable linear regression analysis for SvdH score at MCPJ 2 to 4 in PsA patients. **Supplementary Table 5.** Univariate and multivariable linear regression analysis for JS volume at MCPJ 2 to 4 in PsA patients. **Supplementary Table 6.** Univariate and multivariable linear regression analysis for mean JSW at MCPJ2, MCPJ3, MCPJ4 in PsA patients. **Supplementary Table 7.** Univariate and multivariable linear regression analysis for maximum JSW at MCPJ2, MCPJ3, MCPJ4 in PsA patients. **Supplementary Table 8.** Univariate and multivariable linear regression analysis for minimum JSW at MCPJ2, MCPJ3, MCPJ4 in PsA patients. **Supplementary Table 9.** Univariate and multivariable linear regression analysis for JSW SD at MCPJ2, MCPJ3, MCPJ4 in PsA patients. **Supplementary Table 10.** Univariate and multivariable linear regression analysis for JSW AS at MCPJ2, MCPJ3, MCPJ4 in PsA patients. **Supplementary Table 11.** Multivariable linear regression analysis between demographic and disease-related variables and JSW parameters at MCPJ 2 to 4.

## Data Availability

Data underlying this article are available from the corresponding author upon reasonable request.

## Abbreviations

JS: Joint space
JSW: Joint space width
JSN: Joint space narrowing
HR-pQCT: High-resolution peripheral quantitative computed tomography
PsA: Psoriatic arthritis
MCPJ: Metacarpophalangeal joint
JSV: Joint space volume
csDMARDs: Conventional synthetic disease-modifying anti-rheumatic drugs
bDMARDs: Biologic DMARDs
CRP: C-reactive protein
ESR: Erythrocyte sedimentation rate
DAPSA: Disease Activity in Psoriatic Arthritis
HAQ: Health Assessment Questionnaire score
SD: Standard deviation
Max JSW: Maximum joint space width
Min JSW: Minimum joint space width
Asymm: Joint asymmetry
SvdH: Sharp/van der Heijde
GEE: Generalized estimating equations
CI: Confidence interval
