# Supplementary material for: Role of inflammatory burden and treatment on joint space width in psoriatic arthritis—a high-resolution peripheral quantitative computed tomography study
Source: Arthritis Res Ther. 2023 Aug 3;25:138. doi: 10.1186/s13075-023-03124-5 (PMC10399015; doi:10.1186/s13075-023-03124-5)
Supplement: Supplementary file 1 — Additional file 1: Supplementary Table 1. Joint space analysis using HR-pQCT for PsA patients. Supplementary Table 2. Joint space narrowing scored using the van der Heijde-modified Sharp scoring system on conventional radiographs. Supplementary Table 3. The ability of HR-pQCT joint space analysis to predict SvdH scores on conventional radiography using Generalized estimating equations (GEE). Supplementary Table 4. Univariate and multivariable linear regression analysis for SvdH score at MCPJ 2 to 4 in PsA patients. Supplementary Table 5. Univariate and multivariable linear regression analysis for JS volume at MCPJ 2 to 4 in PsA patients. Supplementary Table 6. Univariate and multivariable linear regression analysis for mean JSW at MCPJ2, MCPJ3, MCPJ4 in PsA patients. Supplementary Table 7. Univariate and multivariable linear regression analysis for maximum JSW at MCPJ2, MCPJ3, MCPJ4 in PsA patients. Supplementary Table 8. Univariate and multivariable linear regression analysis for minimum JSW at MCPJ2, MCPJ3, MCPJ4 in PsA patients. Supplementary Table 9. Univariate and multivariable linear regression analysis for JSW SD at MCPJ2, MCPJ3, MCPJ4 in PsA patients. Supplementary Table 10. Univariate and multivariable linear regression analysis for JSW AS at MCPJ2, MCPJ3, MCPJ4 in PsA patients. Supplementary Table 11. Multivariable linear regression analysis between demographic and disease-related variables and JSW parameters at MCPJ 2 to 4. [file 13075_2023_3124_MOESM1_ESM.docx]

**Supplementary Table 1. Joint space analysis using HR-pQCT for PsA patients**

| JS Parameters | MCPJ 2 | MCPJ 3 | MCPJ 4 |
| --- | --- | --- | --- |
| JS Volume (mm3) | 87.1±18.8 | 94.1±22.7 | 66.1±15.0 |
| Mean JSW (mm) | 1.8±0.2 | 1.7±0.2 | 1.6±0.2 |
| JSW SD (mm) | 0.3 (0.3, 0.3) | 0.3±0.0 | 0.3±0.1 |
| Max JSW (mm) | 2.9 (2.7, 2.9) | 2.7 (2.5, 2.9) | 2.6 (2.5, 2.7) |
| Min JSW (mm) | 1.1(0.8, 1.4) | 1.1 (0.2, 1.3) | 1.0 (0.9, 1.2) |
| JSW Asymm | 2.4(2.1, 3.2) | 2.5 (2.2, 15.8) | 2.4 (2.1, 3.0) |

Values are presented as mean ± SD or median (interquartile range)

Individual MCP joint space parameters.

PsA, psoriatic arthritis; MCPJ, metacarpophalangeal joint; JS, joint space; JSW, joint space width; SD, standard deviation; Max JSW, maximum joint space width; Min JSW, minimum joint space width; Asymm, joint asymmetry.

**Supplementary Table 2. Joint space narrowing scored using the van der Heijde-modified Sharp scoring system on conventional radiographs**

|  | 0 (Normal) | 1 (Focal or Doubtful) | 2 (> 50% of Original JS) | 3 (< 50% of Original JS or Subluxation) | 4 (Bony Ankylosis or Complete Luxation) |
| --- | --- | --- | --- | --- | --- |
| MCPJ 2 | 41 | 0 | 13 | 0 | 2 |
| MCPJ 3 | 41 | 0 | 13 | 3 | 1 |
| MCPJ 4 | 44 | 0 | 12 | 4 | 0 |

MCPJ, metacarpophalangeal joint.

**Supplementary Table 3. The ability of HR-pQCT joint space analysis to predict SvdH scores on conventional radiography using Generalized estimating equations (GEE).**

|  | B | 95% CI | p |
| --- | --- | --- | --- |
| JS Volume (mm^3^) | -0.003 | -0.010, 0.004 | 0.462 |
| Mean JSW (mm) | -1.548 | -2.154, -0.942 | **0.000** |
| JSW SD (mm) | 3.127 | 0.382, 5.872 | **0.026** |
| Max JSW (mm) | -0.960 | -1.673, -0.246 | **0.008** |
| Min JSW (mm) | -0.801 | -1.116, -0.486 | **0.000** |
| JSW Assym | 0.039 | 0.013, 0.066 | **0.004** |

JS, joint space; JSW, joint space width; SD, standard deviation; Max JSW, maximum joint space width; Min JSW, minimum joint space width; Assym, joint asymmetry.

**Supplementary Table 4. Univariate and multivariable linear regression analysis for SvdH score at MCPJ 2 to 4 in PsA patients.**

|  | MCPJ 2 | | | | | MCPJ 3 | | | MCPJ 4 | | |
| --- | --- | --- | --- | --- | --- | --- | --- | --- | --- | --- | --- |
|  | Beta | 95% CI | | p | Beta | | 95% CI | p | Beta | 95% CI | p |
| **Univariate analysis** |  | |  |  |  | |  |  |  |  |  |
| Age | -0.001 | -0.031, 0.030 | | 0.967 | 0.005 | | -0.029, 0.039 | 0.770 | 0.012 | -0.016, 0.041 | 0.380 |
| Sex, female | 0.104 | -0.482, 0.690 | | 0.723 | 0.315 | | -0.265, 0.895 | 0.281 | 0.299 | -0.237, 0.834 | 0.269 |
| Disease duration (years) | 0.017 | -0.014, 0.048 | | 0.283 | 0.026 | | -0.005, 0.058 | 0.099 | 0.015 | -0.012, 0.042 | 0.264 |
| ESR | 0.015 | 0.000, 0.031 | | 0.055 | 0.010 | | -0.006, 0.026 | 0.220 | 0.009 | -0.004, 0.023 | 0.159 |
| CRP | -0.004 | -0.064, 0.056 | | 0.897 | -0.005 | | -0.067, 0.056 | 0.865 | 0.027 | -0.009, 0.062 | 0.136 |
| DAPSA | 0.000 | -0.051, 0.051 | | 0.998 | 0.023 | | -0.024, 0.069 | 0.332 | 0.029 | -0.012, 0.069 | 0.161 |
| HAQ | -0.303 | -0.879, 0.273 | | 0.297 | 0.045 | | -0.493, 0.583 | 0.867 | 0.057 | -0.438, 0.552 | 0.819 |
| Tender joint count | 0.012 | -0.104, 0.128 | | 0.836 | 0.068 | | -0.031, 0.168 | 0.173 | 0.085 | -0.015, 0.186 | 0.095 |
| Swollen joint count | 0.258 | 0.070, 0.446 | | **0.008** | 0.211 | | 0.045, 0.376 | **0.013** | 0.164 | 0.014, 0.314 | **0.033** |
| Damage joint count | 0.064 | 0.031, 0.097 | | **0.000** | 0.054 | | 0.022, 0.085 | **0.001** | 0.043 | 0.013, 0.072 | **0.005** |
| csDMARDs (%) | 0.009 | -0.749, 0.766 | | 0.982 | 0.208 | | -0.561, 0.978 | 0.590 | 0.480 | -0.228, 1.188 | 0.180 |
| bDMARDs (%) | -0.440 | -10.018, 0.137 | | 0.132 | -0.278 | | -0.874, 0.318 | 0.355 | -0.343 | -0.879, 0.193 | 0.206 |

**Multivariate analysis**

|  | MCPJ 2 | | | MCPJ 3 | | | MCPJ 4 | | |
| --- | --- | --- | --- | --- | --- | --- | --- | --- | --- |
|  | Beta | 95% CI | p | Beta | 95% CI | p | Beta | 95% CI | p |
| Swollen joint count | 0.146 | -0.046, 0.337 | 0.133 | 0.132 | -0.035, 0.299 | 0.119 | 0.110 | -0.042, 0.262 | 0.152 |
| Damage joint count | 0.053 | 0.017, 0.089 | **0.004** | 0.045 | 0.012, 0.078 | **0.009** | 0.036 | 0.006, 0.066 | **0.021** |

SvdH, Sharp/van der Heijde; MCPJ, metacarpophalangeal joint; PsA, psoriatic arthritis; ESR, erythrocyte sedimentation rate; CRP, C-reactive protein; DAPSA, Disease Activity in Psoriatic Arthritis; HAQ, Health Assessment Questionnaire score; csDMARDs, conventional synthetic disease-modifying anti-rheumatic drugs; bDMARDs, biologic disease-modifying anti-rheumatic drugs.

**Supplementary Table 5. Univariate and multivariable linear regression analysis for JS volume at MCPJ 2 to 4 in PsA patients.**

|  | MCPJ 2 | | | | | MCPJ 3 | | | MCPJ 4 | | |
| --- | --- | --- | --- | --- | --- | --- | --- | --- | --- | --- | --- |
|  | Beta | 95% CI | | p | Beta | | 95% CI | p | Beta | 95% CI | p |
| **Univariate analysis** |  | |  |  |  | |  |  |  |  |  |
| Age | 0.478 | -0.045, 1.002 | | 0.072 | 0.193 | | -0.511, 0.898 | 0.585 | 0.320 | -0.085, 0.726 | 0.119 |
| Sex, female | -26.826 | -34.023, -19.629 | | **0.000** | -31.281 | | -40.056, -22.505 | **0.000** | -21.618 | -27.118, -16.118 | **0.000** |
| Disease duration (years) | -0.352 | -0.891, 0.187 | | 0.196 | -0.475 | | -1.126, 0.177 | 0.150 | -0.143 | -0.538, 0.252 | 0.471 |
| ESR | -0.196 | -0.477, 0.085 | | 0.167 | -0.310 | | -.638, 0.018 | 0.063 | -0.158 | -0.351, 0.034 | 0.105 |
| CRP | -0.829 | -1.856, 0.198 | | 0.111 | -1.031 | | -2.278, 0.216 | 0.103 | -0.192 | -0.719, 0.336 | 0.470 |
| DAPSA | -0.382 | -1.272, 0.509 | | 0.394 | -0.448 | | -1.407, 0.511 | 0.353 | -0.184 | -0.781, 0.413 | 0.539 |
| HAQ | -1.273 | -11.452, 8.907 | | 0.803 | -0.913 | | -12.052, 10.226 | 0.870 | -0.666 | -7.905, 6.574 | 0.855 |
| Tender joint count | -0.697 | -2.720, 1.326 | | 0.493 | -0.628 | | -2.708, 1.453 | 0.548 | -1.492 | -2.946, -0.038 | **0.044** |
| Swollen joint count | -1.284 | -4.783, 2.215 | | 0.465 | -1.009 | | -4.613, 2.595 | 0.577 | -1.054 | -3.322, 1.213 | 0.356 |
| Damage joint count | 0.559 | -0.082, 1.201 | | 0.086 | 0.374 | | -0.336, 1.084 | 0.296 | 0.193 | -0.260, 0.647 | 0.396 |
| csDMARDs (%) | -0.977 | -14.238, 12.283 | | 0.883 | -5.684 | | -21.581, 10.213 | 0.477 | -1.624 | -12.131, 8.882 | 0.758 |
| bDMARDs (%) | -0.424 | -10.749, 9.901 | | 0.935 | 3.708 | | -8.685, 16.100 | 0.551 | 3.296 | -4.605, 11.198 | 0.407 |

**Multivariate analysis**

|  | | MCPJ 2 | | | MCPJ 3 | | | | MCPJ 4 | | |
| --- | --- | --- | --- | --- | --- | --- | --- | --- | --- | --- | --- |
|  | Beta | | 95% CI | p | Beta | 95% CI | p | Beta | | 95% CI | p |
| Sex, female | -26.826 | | -34.023, -19.629 | **0.000** | -31.281 | -40.056, -22.505 | **0.000** | -20.141 | | -26.133, -14.149 | **0.000** |
| Tender joint count | - | | - | **-** | **-** | **-** | **-** | -0.398 | | -1.538, 0.743 | 0.488 |

MCPJ, metacarpophalangeal joint; PsA, psoriatic arthritis; JSW, joint space width; AS, joint asymmetry; ESR, erythrocyte sedimentation rate; CRP, C-reactive protein; DAPSA, Disease Activity in Psoriatic Arthritis; HAQ, Health Assessment Questionnaire score; csDMARDs, conventional synthetic disease-modifying anti-rheumatic drugs; bDMARDs, biologic disease-modifying anti-rheumatic drugs.

**Supplementary Table** **6. Univariate and multivariable linear regression analysis for mean JSW at MCPJ2, MCPJ3, MCPJ4 in PsA patients.**

|  | MCPJ 2 | | | | MCPJ 3 | | | MCPJ 4 | | |
| --- | --- | --- | --- | --- | --- | --- | --- | --- | --- | --- |
|  | Beta | 95% CI | | p | Beta | 95% CI | p | Beta | 95% CI | p |
| **Univariate analysis** |  | |  |  |  |  |  |  |  |  |
| Age | 0.000 | -0.006, 0.006 | | 0.951 | -0.003 | -0.010, 0.005 | 0.479 | 0.002 | -0.004, 0.009 | 0.461 |
| Sex, female | -0.092 | -0.206, 0.022 | | 0.113 | -0.171 | -0.287, -0.054 | **0.005** | -0.135 | -0.250, -0.019 | **0.023** |
| Disease duration (years) | -0.009 | -0.015, -0.004 | | **0.002** | -0.011 | -0.017, -0.005 | **0.001** | -0.004 | -0.010, 0.002 | 0.176 |
| ESR | -0.004 | -0.007, -0.001 | | **0.014** | -0.006 | -0.009, -0.002 | **0.001** | -0.004 | -0.006, -0.001 | **0.014** |
| CRP | -0.017 | -0.028, -0.006 | | **0.003** | -0.020 | -0.032, -0.008 | **0.001** | -0.011 | -0.018, -0.003 | **0.006** |
| DAPSA | -0.007 | -0.017, 0.003 | | 0.146 | -0.008 | -0.018, 0.001 | 0.094 | -0.007 | -0.016, 0.002 | 0.139 |
| HAQ | 0.015 | -0.101, 0.131 | | 0.799 | -0.001 | -0.116, 0.113 | 0.984 | 0.014 | -0.097, 0.125 | 0.801 |
| Tender joint count | -0.008 | -0.031, 0.016 | | 0.516 | -0.015 | -0.036, 0.006 | 0.157 | -0.018 | -0.041, 0.004 | 0.107 |
| Swollen joint count | -0.052 | -0.089, -0.014 | | **0.008** | -0.038 | -0.074, -0.002 | **0.038** | -0.042 | -0.075, -0.009 | **0.015** |
| Damage joint count | -0.011 | -0.018, -0.005 | | **0.002** | -0.010 | -0.017, -0.003 | **0.007** | -0.008 | -0.015, -0.001 | **0.018** |
| csDMARDs (%) | -0.083 | -0.232, 0.066 | | 0.270 | -0.212 | -0.366, -0.058 | **0.008** | -0.079 | -0.238, 0.080 | 0.325 |
| bDMARDs (%) | 0.075 | -0.041, 0.191 | | 0.201 | 0.052 | -0.076, 0.179 | 0.419 | 0.044 | -0.077, 0.165 | 0.468 |

**Multivariate analysis**

|  | MCPJ 2 | | | MCPJ 3 | | | MCPJ 4 | | |
| --- | --- | --- | --- | --- | --- | --- | --- | --- | --- |
|  | Beta | 95% CI | p | Beta | 95% CI | p | Beta | 95% CI | p |
| **Model 1** |  |  |  |  |  |  |  |  |  |
| Sex, female | - | - | - | -0.104 | -0.209, 0.000 | 0.051 | -0.119 | -0.232, -0.006 | **0.039** |
| Disease duration (years) | -0.007 | -0.013, -0.002 | **0.008** | -0.008 | -0.013, -0.002 | **0.011** | - | - | - |
| ESR | -0.002 | -0.005, 0.001 | 0.191 | -0.004 | -0.007, 0.000 | **0.028** | -0.002 | -0.005, 0.001 | 0.246 |
| CRP |  | | |  | | |  | | |
| Swollen joint count | -0.014 | -0.053, 0.026 | 0.489 | 0.008 | -0.026, 0.042 | 0.638 | -0.015 | -0.052, 0.022 | 0.433 |
| Damage joint count | -0.008 | -0.015, -0.001 | **0.034** | -0.005 | -0.011, 0.001 | 0.117 | -0.006 | -0.013, 0.000 | 0.062 |
| DMARDs (%) | - | - | - | -0.126 | -0.259, 0.008 | 0.064 | - | - | - |
| **Model 2** |  |  |  |  |  |  |  |  |  |
| Sex, female | - | - | - | -0.109 | -0.217, -0.002 | **0.047** | -0.129 | -0.239, -0.019 | **0.023** |
| Disease duration (years) | -0.007 | -0.013, -0.002 | **0.010** | -0.007 | -0.013, -0.001 | **0.019** | - | - | - |
| ESR |  | | |  | | |  | | |
| CRP | -0.008 | -0.020, 0.004 | 0.167 | -0.009 | -0.022, 0.004 | 0.157 | -0.008 | -0.015, 0.000 | 0.056 |
| Swollen joint count | -0.016 | -0.054, 0.022 | 0.410 | 0.004 | -0.031, 0.039 | 0.824 | -0.014 | -0.049, 0.020 | 0.405 |
| Damage joint count | -0.007 | -0.014, 0.000 | 0.058 | -0.005 | -0.012, 0.002 | 0.147 | -0.005 | -0.012, 0.001 | 0.111 |
| csDMARDs (%) | - | - | - | -0.142 | -0.278, -0.005 | **0.042** | - | - | - |

MCPJ, metacarpophalangeal joint; PsA, psoriatic arthritis; JSW, joint space width; AS, joint asymmetry; ESR, erythrocyte sedimentation rate; CRP, C-reactive protein; DAPSA, Disease Activity in Psoriatic Arthritis; HAQ, Health Assessment Questionnaire score; csDMARDs, conventional synthetic disease-modifying anti-rheumatic drugs; bDMARDs, biologic disease-modifying anti-rheumatic drugs.

**Supplementary Table 7. Univariate and multivariable linear regression analysis for maximum JSW at MCPJ2, MCPJ3, MCPJ4 in PsA patients.**

|  | MCPJ 2 | | | | | MCPJ 3 | | | MCPJ 4 | | |
| --- | --- | --- | --- | --- | --- | --- | --- | --- | --- | --- | --- |
|  | Beta | | 95% CI | p | Beta | | 95% CI | p | Beta | 95% CI | p |
| **Univariate analysis** |  |  | |  |  | |  |  |  |  |  |
| Age | 0.003 | | -0.002, 0.008 | 0.191 | -0.001 | | -0.007, 0.004 | 0.622 | 0.004 | -0.003, 0.010 | 0.262 |
| Sex, female | -0.076 | | -0.164, 0.012 | 0.091 | -0.144 | | -0.233, -0.056 | **0.002** | -0.070 | -0.190, 0.050 | 0.247 |
| Disease duration (years) | -0.006 | | -0.011, -0.002 | **0.008** | -0.008 | | -0.013, -0.003 | **0.002** | -0.002 | -0.008, 0.004 | 0.441 |
| ESR | -0.002 | | -0.005, 0.000 | 0.095 | -0.004 | | -0.006, -0.001 | **0.002** | -0.001 | -0.004, 0.002 | 0.418 |
| CRP | -0.015 | | -0.024, -0.007 | **0.000** | -0.012 | | -0.022, -0.002 | **0.016** | -0.008 | -0.016, 0.000 | **0.047** |
| DAPSA | 0.002 | | -0.006, 0.010 | 0.629 | -0.003 | | -0.010, 0.005 | 0.486 | -0.002 | -0.011, 0.007 | 0.709 |
| HAQ | 0.025 | | -0.065, 0.115 | 0.581 | 0.008 | | -0.081, 0.097 | 0.863 | 0.022 | -0.089, 0.132 | 0.697 |
| Tender joint count | 0.003 | | -0.015, 0.021 | 0.742 | -0.004 | | -0.021, 0.012 | 0.617 | -0.002 | -0.025, 0.021 | 0.853 |
| Swollen joint count | -0.017 | | -0.048, 0.014 | 0.266 | -0.031 | | -0.058, -0.003 | **0.029** | -0.025 | -0.060, 0.009 | 0.145 |
| Damage joint count | -0.004 | | -0.010, 0.002 | 0.175 | -0.003 | | -0.009, 0.002 | 0.225 | -0.006 | -0.013, 0.001 | 0.091 |
| csDMARDs (%) | -0.058 | | -0.174, 0.059 | 0.324 | -0.075 | | -0.201, 0.051 | 0.236 | -0.031 | -0.192, 0.130 | 0.699 |
| bDMARDs (%) | 0.040 | | -0.051, 0.131 | 0.381 | 0.028 | | -0.071, 0.127 | 0.571 | 0.067 | -0.053, 0.187 | 0.270 |

**Multivariate analysis**

|  | MCPJ 2 | | | MCPJ 3 | | | MCPJ 4 | | |
| --- | --- | --- | --- | --- | --- | --- | --- | --- | --- |
|  | Beta | 95% CI | p | Beta | 95% CI | p | Beta | 95% CI | p |
| **Model 1** |  |  |  |  |  |  |  |  |  |
| Sex, female | - | - | - | -0.098 | -0.185, -0.011 | **0.029** | - | - | - |
| Disease duration (years) | -0.006 | -0.010, -0.002 | **0.005** | -0.005 | -0.010, -0.001 | **0.029** | - | - | - |
| ESR | - | - | - | -0.003 | -0.005, 0.000 | **0.027** | - | - | - |
| CRP | -0.014 | -0.022, -0.006 | **0.001** |  | | | -0.008 | -0.016, 0.000 | **0.047** |
| Swollen joint count | - | - | - | -0.004 | -0.032, 0.024 | 0.773 | - | - | - |
| **Model 2** |  |  |  |  |  |  | - | - | - |
| Sex, female | - | - | - | -0.104 | -0.194, -0.014 | **0.024** | - | - | - |
| Disease duration (years) | - | - | - | -0.005 | -0.010, 0.000 | **0.044** | - | - | - |
| ESR | - | - | - |  | | |  |  | - |
| CRP | - | - | - | -0.006 | -0.017, 0.004 | 0.219 | - | - | - |
| Swollen joint count | - | - | - | -0.009 | -0.037, 0.020 | 0.538 | - | - | - |

MCPJ, metacarpophalangeal joint; PsA, psoriatic arthritis; JSW, joint space width; AS, joint asymmetry; ESR, erythrocyte sedimentation rate; CRP, C-reactive protein; DAPSA, Disease Activity in Psoriatic Arthritis; HAQ, Health Assessment Questionnaire score; csDMARDs, conventional synthetic disease-modifying anti-rheumatic drugs; bDMARDs, biologic disease-modifying anti-rheumatic drugs.

**Supplementary Table 8. Univariate and multivariable linear regression analysis for minimum JSW at MCPJ2, MCPJ3, MCPJ4 in PsA patients.**

|  | MCPJ 2 | | | | | MCPJ 3 | | | MCPJ 4 | | |
| --- | --- | --- | --- | --- | --- | --- | --- | --- | --- | --- | --- |
|  | Beta | 95% CI | | p | Beta | | 95% CI | p | Beta | 95% CI | p |
| **Univariate analysis** |  | |  |  |  | |  |  |  |  |  |
| Age | 0.002 | -0.012, 0.015 | | 0.820 | -0.010 | | -0.026, 0.007 | 0.244 | -0.001 | -0.011, 0.009 | 0.835 |
| Sex, female | 0.098 | -0.165, 0.361 | | 0.457 | -0.057 | | -0.344, 0.230 | 0.693 | -0.200 | -0.376, -0.024 | **0.026** |
| Disease duration (years) | 0.004 | -0.010, 0.018 | | 0.541 | -0.001 | | -0.017, 0.015 | 0.908 | -0.005 | -0.014, 0.004 | 0.252 |
| ESR | -0.003 | -0.010, 0.005 | | 0.480 | -0.010 | | -0.018, -0.002 | **0.011** | -0.006 | -0.010, -0.002 | **0.007** |
| CRP | -0.011 | -0.038, 0.015 | | 0.397 | -0.026 | | -0.055, 0.004 | 0.087 | -0.019 | -0.030, -0.008 | **0.001** |
| DAPSA | -0.019 | -0.042, 0.003 | | 0.088 | -0.015 | | -0.038, 0.008 | 0.187 | -0.021 | -0.033, -0.008 | **0.002** |
| HAQ | -0.017 | -0.279, 0.245 | | 0.899 | 0.087 | | -0.176, 0.350 | 0.511 | -0.153 | -0.316, 0.010 | 0.065 |
| Tender joint count | -0.011 | -0.063, 0.041 | | 0.680 | -0.019 | | -0.069, 0.030 | 0.432 | -0.047 | -0.080, -0.014 | **0.006** |
| Swollen joint count | -0.062 | -0.151, 0.027 | | 0.168 | -0.057 | | -0.142, 0.027 | 0.179 | -0.070 | -0.120, -0.020 | **0.007** |
| Damage joint count | -0.016 | -0.033, 0.000 | | **0.049** | -0.013 | | -0.030, 0.003 | 0.116 | -0.015 | -0.025, -0.005 | **0.004** |
| csDMARDs (%) | -0.176 | -0.514, 0.162 | | 0.300 | -0.412 | | -0.774, -0.049 | **0.027** | -0.220 | -0.457, 0.017 | 0.068 |
| bDMARDs (%) | 0.279 | 0.025, 0.534 | | **0.032** | 0.393 | | 0.117, 0.668 | **0.006** | 0.002 | -0.182, 0.186 | 0.984 |

**Multivariate analysis**

|  | MCPJ 2 | | | MCPJ 3 | | | MCPJ 4 | | |
| --- | --- | --- | --- | --- | --- | --- | --- | --- | --- |
|  | Beta | 95% CI | p | Beta | 95% CI | p | Beta | 95% CI | p |
| **Model 1** |  |  |  |  |  |  |  |  |  |
| Sex, female | - | - | - | - | - | - | -0.138 | -0.309, 0.034 | 0.113 |
| ESR | - | - | - | -0.006 | -0.014, 0.001 | 0.098 | -0.003 | -0.007, 0.002 | 0.235 |
| CRP | - | - | - | - | - | - |  | | |
| DAPSA | - | - | - | - | - | - | -0.006 | -0.024, 0.011 | 0.456 |
| Tender joint count | - | - | - | - | - | - | -0.016 | -0.057, 0.024 | 0.422 |
| Swollen joint count | - | - | - | - | - | - | -0.010 | -0.068, 0.047 | 0.720 |
| Damage joint count | -0.016 | -0.032, 0.000 | **0.049** | - | - | - | -0.011 | -0.021, -0.001 | **0.034** |
| csDMARDs (%) | - | - | - | -0.389 | -0.728, -0.049 | **0.026** | - | - | - |
| bDMARDs (%) | 0.271 | 0.023, 0.519 | **0.033** | 0.366 | 0.100, 0.631 | **0.008** | - | - | - |
| **Model 2** |  |  |  |  |  |  |  |  |  |
| Sex, female | - | - | - | - | - | - | -0.156 | -0.322, 0.010 | 0.064 |
| ESR | - | - | - | - | - | - |  | | |
| CRP | - | - | - | - | - | - | -0.015 | -0.028, -0.001 | **0.031** |
| DAPSA | - | - | - | - | - | - | 0.003 | -0.016, 0.023 | 0.735 |
| Tender joint count | - | - | - | - | - | - | -0.031 | -0.073, 0.011 | 0.146 |
| Swollen joint count | - | - | - | - | - | - | -0.017 | -0.070, 0.035 | 0.510 |
| Damage joint count | - | - | - | - | - | - | -0.009 | -0.019, 0.001 | 0.086 |
| csDMARDs (%) | - | - | - | - | - | - | - | - | - |
| bDMARDs (%) | - | - | - | - | - | - | - | - | - |

MCPJ, metacarpophalangeal joint; PsA, psoriatic arthritis; JSW, joint space width; AS, joint asymmetry; ESR, erythrocyte sedimentation rate; CRP, C-reactive protein; DAPSA, Disease Activity in Psoriatic Arthritis; HAQ, Health Assessment Questionnaire score; csDMARDs, conventional synthetic disease-modifying anti-rheumatic drugs; bDMARDs, biologic disease-modifying anti-rheumatic drugs.

**Supplementary Table 9. Univariate and multivariable linear regression analysis for JSW SD at MCPJ2, MCPJ3, MCPJ4 in PsA patients.**

|  | MCPJ 2 | | | | | | MCPJ 3 | | | MCPJ 4 | | |
| --- | --- | --- | --- | --- | --- | --- | --- | --- | --- | --- | --- | --- |
|  | Beta | 95% CI | | p | | Beta | | 95% CI | p | Beta | 95% CI | p |
| **Univariate analysis** |  | |  |  | |  | |  |  |  |  |  |
| Age | 0.000 | -0.002, 0.002 | | 0.767 | | 0.000 | | -0.001, 0.002 | 0.860 | 0.001 | -0.001, 0.002 | 0.370 |
| Sex, female | 0.006 | -0.031, 0.044 | | 0.744 | | 0.000 | | -0.025, 0.025 | 0.980 | 0.020 | -0.007, 0.046 | 0.148 |
| Disease duration (years) | 0.000 | -0.002, 0.002 | | 0.718 | | -0.001 | | -0.002, 0.000 | 0.201 | 0.000 | -0.001, 0.002 | 0.713 |
| ESR | 0.000 | -0.001, 0.001 | | 0.679 | | 0.000 | | -0.001, 0.000 | 0.291 | 0.000 | 0.000, 0.001 | 0.159 |
| CRP | -0.001 | -0.005, 0.002 | | 0.449 | | -0.002 | | -0.004, 0.001 | 0.213 | 0.001 | -0.001, 0.002 | 0.485 |
| DAPSA | 0.002 | -0.001, 0.005 | | 0.244 | | 0.001 | | -0.001, 0.003 | 0.147 | 0.001 | -0.001, 0.003 | 0.148 |
| HAQ | -0.012 | -0.049, 0.025 | | 0.522 | | 0.015 | | -0.008, 0.037 | 0.197 | 0.028 | 0.004, 0.052 | **0.023** |
| Tender joint count | 0.003 | -0.004, 0.011 | | 0.393 | | 0.003 | | -0.002, 0.007 | 0.219 | 0.003 | -0.002, 0.008 | 0.307 |
| Swollen joint count | 0.009 | -0.003, 0.022 | | 0.145 | | 0.001 | | -0.006, 0.009 | 0.715 | 0.004 | -0.004, 0.012 | 0.303 |
| Damage joint count | 0.000 | -0.002, 0.002 | | 0.967 | | 0.001 | | -0.001, 0.002 | 0.440 | 0.000 | -0.002, 0.001 | 0.920 |
| csDMARDs (%) | -0.030 | -0.078, 0.018 | | | 0.217 | 0.003 | | -0.030, 0.036 | 0.848 | 0.010 | -0.026, 0.046 | 0.582 |
| bDMARDs (%) | -0.012 | -0.050, 0.026 | | 0.523 | | 0.002 | | -0.024, 0.027 | 0.901 | -0.009 | -0.036, 0.018 | 0.505 |

**Multivariate analysis**

|  | MCPJ 2 | | | MCPJ 3 | | | MCPJ 4 | | |
| --- | --- | --- | --- | --- | --- | --- | --- | --- | --- |
|  | Beta | 95% CI | p | Beta | 95% CI | p | Beta | 95% CI | p |
| HAQ | - | - | - | - | - | - | 0.028 | 0.004, 0.052 | **0.023** |

MCPJ, metacarpophalangeal joint; PsA, psoriatic arthritis; JSW, joint space width; AS, joint asymmetry; ESR, erythrocyte sedimentation rate; CRP, C-reactive protein; DAPSA, Disease Activity in Psoriatic Arthritis; HAQ, Health Assessment Questionnaire score; csDMARDs, conventional synthetic disease-modifying anti-rheumatic drugs; bDMARDs, biologic disease-modifying anti-rheumatic drugs.

**Supplementary Table 10. Univariate and multivariable linear regression analysis for JSW AS at MCPJ2, MCPJ3, MCPJ4 in PsA patients.**

|  | MCPJ 2 | | | | | MCPJ 3 | | | MCPJ 4 | | |
| --- | --- | --- | --- | --- | --- | --- | --- | --- | --- | --- | --- |
|  | Beta | 95% CI | | p | Beta | | 95% CI | p | Beta | 95% CI | p |
| **Univariate analysis** |  | |  |  |  | |  |  |  |  |  |
| Age | -0.034 | -0.202, 0.134 | | 0.685 | 0.119 | | -0.090, 0.329 | 0.257 | 0.024 | -0.079, 0.127 | 0.641 |
| Sex, female | -2.521 | -5.647, 0.606 | | 0.112 | -1.146 | | -4.764, 2.471 | 0.528 | 0.725 | -1.229, 2.678 | 0.461 |
| Disease duration (years) | -0.159 | -0.324, 0.006 | | 0.059 | -0.080 | | -0.275, 0.115 | 0.416 | 0.021 | -0.078, 0.120 | 0.678 |
| ESR | -0.010 | -0.099, 0.079 | | 0.818 | 0.107 | | 0.010, 0.204 | **0.031** | 0.050 | 0.003, 0.097 | **0.039** |
| CRP | -0.046 | -0.374, 0.282 | | 0.779 | 0.276 | | -0.099, 0.651 | 0.146 | 0.167 | 0.043, 0.291 | **0.009** |
| DAPSA | 0.126 | -0.151, 0.404 | | 0.365 | 0.125 | | -0.162, 0.412 | 0.388 | 0.191 | 0.051, 0.331 | **0.008** |
| HAQ | 0.332 | -2.843, 3.507 | | 0.835 | -1.458 | | -4.770, 1.854 | 0.382 | 1.814 | 0.080, 3.547 | **0.041** |
| Tender joint count | -0.057 | -0.690, 0.577 | | 0.858 | 0.087 | | -0.537, 0.712 | 0.781 | 0.447 | 0.092, 0.801 | **0.015** |
| Swollen joint count | 0.110 | -0.986, 1.207 | | 0 .841 | 0.450 | | -0.625, 1.525 | 0.406 | 0.513 | -0.038, 1.064 | 0.067 |
| Damage joint count | 0.067 | -0.138, 0.272 | | 0.513 | 0.072 | | -0.142, 0.286 | 0.503 | 0.091 | -0.019, 0.202 | 0.104 |
| csDMARDs (%) | 0.069 | -4.068, 4.205 | | 0.974 | 2.906 | | -1.811, 7.622 | 0.222 | 1.605 | -0.971, 4.181 | 0.217 |
| bDMARDs (%) | -2.503 | -5.650, 0.644 | | 0.117 | -5.410 | | -8.838, -1.982 | **0.003** | 0.711 | -1.254, 2.675 | 0.472 |

**Multivariate analysis**

|  | MCPJ 2 | | | MCPJ 3 | | | MCPJ 4 | | |
| --- | --- | --- | --- | --- | --- | --- | --- | --- | --- |
|  | Beta | 95% CI | p | Beta | 95% CI | p | Beta | 95% CI | p |
| **Model 1** |  |  |  |  |  |  |  |  |  |
| ESR | - | - | - | 0.079 | -.015, 0.173 | 0.099 | 0.035 | -0.014, 0.084 | 0.158 |
| CRP |  |  |  |  |  |  |  | | |
| DAPSA | - | - | - | - | - | - | 0.071 | -0.137, 0.279 | 0.496 |
| HAQ | - | - | - | - | - | - | 0.396 | -1.808, 2.600 | 0.720 |
| Tender joint count | - | - | - | - | - | - | 0.251 | -0.222, 0.725 | 0.292 |
| bDMARDs (%) | - | - | - | -4.776 | -8.234 -1.317 | **0.008** | - | - | - |
| **Model 2** |  |  |  |  |  |  |  |  |  |
| ESR | - | - | - | - | - | - |  | | |
| CRP |  |  |  | - | - | - | 0.166 | 0.016, 0.317 | **0.031** |
| DAPSA | - | - | - | - | - | - | -0.036 | -0.274, 0.201 | 0.761 |
| HAQ | - | - | - | - | - | - | 0.711 | -1.460, 2.882 | 0.514 |
| Tender joint count | - | - | - | - | - | - | 0.393 | -0.094, 0.880 | 0.111 |
| bDMARDs (%) | - | - | - | - | - | - | - | - | - |

MCPJ, metacarpophalangeal joint; PsA, psoriatic arthritis; JSW, joint space width; AS, joint asymmetry; ESR, erythrocyte sedimentation rate; CRP, C-reactive protein; DAPSA, Disease Activity in Psoriatic Arthritis; HAQ, Health Assessment Questionnaire score; csDMARDs, conventional synthetic disease-modifying anti-rheumatic drugs; bDMARDs, biologic disease-modifying anti-rheumatic drugs.

**Supplementary Table 11. Multivariable linear regression analysis between demographic and disease-related variables and JSW parameters at MCPJ 2 to 4.**

|  | JS Volume  (beta, 95% CI, p) | Mean JSW  (beta, 95% CI, p) | Max JSW  (beta, 95% CI, p) | Min JSW  (beta, 95% CI, p) | JSW AS  (beta, 95% CI, p) | | JSW SD  (beta, 95% CI, p) | |
| --- | --- | --- | --- | --- | --- | --- | --- | --- |
| **Sex, female** |  |  |  |  |  | |  | |
| MCPJ 2 | -26.826 (-34.023, -19.629), 0.000 | - | - | - | - | | - | |
| MCPJ 3 | -31.281 (-40.056, -22.505), 0.000 | - | -0.098 (-0.185, -0.011), 0.029 | - | - | | - | |
| MCPJ 4 | -20.141 (-26.133, -14.149), 0.000 | -0.119 (-0.232, -0.006), 0.039 | - | - | - | | - | |
| **Disease duration (years)** | | | | | | | | |
| MCPJ 2 | - | -0.007 (-0.013, -0.002), 0.008 | -0.006 (-0.011, -0.002), 0.008 | - | - | | - | |
| MCPJ 3 | - | -0.008 (-0.013, -0.002), 0.011 | -0.005 (-0.010, -0.001), 0.029 | - | - | | - | |
| **ESR (mm/h)** |  |  |  |  |  | |  | |
| MCPJ 3 | - | -0.004 (-0.007, 0.000), 0.028 | -0.003 (-0.005, 0.000), 0.027 | - | - |  | |  |
| **HAQ** |  |  |  |  |  | |  | |
| MCPJ 4 | - | - | - | - | - | | 0.028 (0.004, 0.052), 0.023 | |
| **Damage joint count** | | | | | | | | |
| MCPJ 2 | - | -0.008 (-0.015, -0.001), 0.034 | - | -0.016 (-0.032, 0.000), 0.049 | - | | - | |
| MCPJ 4 | - | - | - | -0.011 (-0.021, -0.001), 0.034 | - | | - | |
| **csDMARDs (%)** |  |  |  |  |  | |  | |
| MCPJ 3 | - | - | - | -0.389 (-0.728, -0.049), 0.026 | - | | - | |
| **bDMARDs (%)** |  |  |  |  |  | |  | |
| MCPJ 2 | - | - | - | 0.271 (0.023, 0.519), 0.033 | - | | - | |
| MCPJ 3 | - | - | - | 0.366 (0.100, 0.631), 0.008 | -4.776 (-8.234, -1.317), 0.008 | | - | |

MCPJ, metacarpophalangeal joint; JS, joint space; JSW, joint space width; SD, standard deviation; Max JSW, maximum joint space width; Minimum JSW, minimum joint space width; AS, joint asymmetry; ESR, erythrocyte sedimentation rate; DAPSA, Disease Activity in Psoriatic Arthritis; HAQ, Health Assessment Questionnaire score; csDMARDs, conventional synthetic disease-modifying anti-rheumatic drugs; bDMARDs, biologic disease-modifying anti-rheumatic drugs.
